# Effects of chronic low-frequency pulsed magnetic fields exposure on the contractility and morphology of biceps brachii in healthy adults–a randomized controlled, double-blind trial

**DOI:** 10.3389/fmed.2025.1614054

**Published:** 2025-07-21

**Authors:** Zhongshan Li, Wenhao Li, Shi Bai, Tieli Yang

**Affiliations:** ^1^School of Sports Science, Fujian Normal University, Fuzhou, Fujian, China; ^2^Department of Physical Education, Northeastern University, Shenyang, Liaoning, China; ^3^School of Information Science and Engineering, Shenyang University of Technology, Shenyang, Liaoning, China

**Keywords:** low frequency pulse magnetic fields, TRPC 1, skeletal muscle morphological structure, muscle strength, cross-transfer

## Abstract

**Background:**

Low-frequency pulse magnetic fields (PEMF) has been proven by classic transient receptor potential channel 1 (TRPC 1) transcription activation peroxisome proliferator-activated receptor-γ coactivator-1α (PGC-1α) increase upstream of the mitochondria calcium - axis to increase muscle and mitochondria function, and recreates the consistent with exercise induced metabolic adaptations and power to ascend.

**Methods:**

Eighty healthy subjects with a mean age of 20 years were recruited and randomly divided into a PEMF group receiving magnetic field stimulation and a control group receiving sham treatment, with 40 patients in each group. The trial lasted for 4 weeks. Both groups were subjected to either a 15 min magnetic stimulation intervention or a sham treatment every 48 h. B-mode ultrasound images were used to evaluate changes in muscle thickness, cross-sectional area, pennation angle, and stiffness.

**Results:**

The PEMF group showed a significant increase in the MVC (maximum voluntary contraction) value and a significantly higher amplitude of the increase than the control group. There was no significant difference in BMI (Body Mass Index) between the two groups before and after the trial, nor in the rate of change in BMI between the two groups. However, from the analysis of the change trends, the PEMF group showed a downward trend, while the control group showed an increasing trend. In the PEMF group, all four parameters of muscle morphology were significantly higher than pre-test, while in the control group, all but one of the muscle stiffness metrics were significantly lower. The PEMF group had significantly higher muscle morphological parameters than did the control group.

**Conclusion:**

The current clinical trial showed that in healthy individuals aged 20 years, after 4 weeks of chronic exposure to a low-frequency pulsed magnetic field (1.5 mT, 3,300 Hz), the maximum voluntary contraction force of the biceps brachii muscle was significantly increased, and a contralateral effect was observed. At the same time, the muscle thickness, cross-sectional area, pennation, and stiffness of the intervention arm increased significantly. It provides a supportive basis for the improvement of muscle histopathology using this technique as an exercise replacement and a medical strategy for muscle improvement.

**Clinical trial registration:**

[https://www.chictr.org.cn], identifier: [ChiCTR2300078947].

## 1 Introduction

As a subtype of electromagnetic fields, low-frequency pulsed electromagnetic fields (PEMF) are an effective and safe magnetic stimulation treatment strategy (1). At the same time, non-thermal biological effects have been the focus of research on biological effects induced by a magnetic field (2). As key magnetic stimulation parameters and associated receptors that play critical roles in the generation of biological effects by pulsed magnetic fields continue to be identified, deeper insights and novel perspectives into the physiological mechanisms underlying magnetic biological effects have been revealed. In recent years, the application of low-frequency PEMF as a clinical strategy for skeletal muscle function and systemic health promotion has become a hot topic in magnetic biomedical research. As an innovative medical-engineering fusion technology for health promotion, Alfredo Franco-obregón’s team found that low-frequency pulsed magnetic fields stimulation with specific parameters (1.5 mT, 3,300 Hz) can mimic the cell response signals induced by mechanical motion. This effect is mediated through the activation of transient receptor potential canonical channel 1 (TRPC 1). Furthermore, the upregulation of peroxisome proliferator-activated receptor-γ coactivator-1α (PGC-1α) transcription via the TRPC 1 pathway modulates the upstream calcium-mitochondrial axis, thereby enhancing mitochondrial function and promoting protein synthesis in muscle tissue (3). The team showed that short-term magnetic stimulation of low-frequency PEMF with specific parameters can facilitate proliferation, repair, and ATP synthesis, enhance mitochondrial respiration, increase skeletal muscle contractility, and promote systemic metabolic levels through improved skeletal muscle function (4–8). Based on this mechanism, the research team also observed that magnetic stimulation could improve the maximum voluntary contractile force and strength endurance of local muscles of the limbs, and the effects of muscle strength maintenance and decay are consistent with resistance training (9, 10). Furthermore, magnetic stimulation significantly improved the lower limb muscle strength of patients with Covid-19 infection during recovery (11). PEMF could be an effective novel strategy for improving skeletal muscle strength and metabolism because of its significant effects on promoting and improving skeletal muscle function.

The improvement of skeletal muscle strength is based on two physiological mechanisms, muscle morphological adaptation, such as changes in the cross-sectional area (CSA), pennation angle (PA), muscle thickness (MT), muscle stiffness (MS), and fiber type of muscle, and neuromuscular adaptation, including muscle fiber recruitment, force development rate, and motor unit rate coding (12). While skeletal muscle structure and morphology are the basis of muscle capacity to generate force, skeletal muscle morphology assessment is a prerequisite for examining functional improvement, regardless of the field of exercise training or medicine for muscle recovery and related diseases (13). Therefore, it is imperative to investigate how low-frequency PEMF stimulation changes muscle morphology. From the perspective of morphological influence, the current study used low-frequency PEMF to activate calcium permeability transient receptor potential TRP channels and found that mesenchymal stem cell secretory molecules and cell-derived vesicles (CDVS) can promote myogenesis in the study of muscle improvement effects in Southeast Asian older adults, and observed strength-related motor function improvement with a significant increase in lean body mass as morphological support. Although there is a positive linear relationship between lean mass and strength gain, changes in lean mass are affected by other factors, including bone and hoof tissue, skeletal muscle mass (4, 14), and enhancement of ligaments such as tendons, which also impacts strength (15). However, the increase in lean body mass is inevitably more dependent on hypertrophy of skeletal muscle, although it is somewhat macroscopical that using the change in lean mass as a support factor for measuring the morphology of skeletal muscle strength improvement, it provides the possibility for this study to evaluate the morphological changes in skeletal muscle by low-frequency PEMF stimulation (16).

Currently, the non-invasive evaluation of skeletal muscle morphological structure mainly depends on dual-energy X-ray absorber (DXA), computed tomography (CT), magnetic resonance imaging (MRI), and brightness mode ultrasound. DXA, CT, and MRI are often used to assess and determine muscle mass (17, 18). However, high cost, poor portability, and high professional requirements limit the application of these technologies in studies with large samples (19). Brightness mode ultrasound imaging has become the primary technique used for diagnosing and evaluating muscle diseases and exercise effects (20). Its technical advantages include good portability, low cost, easy operation, high efficiency, internal consistency of collection indicators, and a consensus gold standard for collection sites (21). They can also be used as references for operating procedures and standards that are suitable for studies with large samples (20, 22–26). In the current study, the biceps brachii of healthy adults were subjected to a low-frequency PEMF for 4 weeks, The changes in MVC of local muscle groups and the effect of muscle morphological structure after force changes were evaluated using brightness mode ultrasound imaging.

## 2 Materials and methods

### 2.1 Subject

According to the grouping and statistical planning of this trial, G-power was used to set the test statistics (two tails; effect size = 0.7; α = 0.05; 1–β = 0.8), after calculation, the sample size was estimated to require 68 subjects, considering the 15% dropout rate. Eighty subjects were recruited for this study. All subjects were freshmen and sophomores from the Bohai Campus of Dalian Oceanic University, with an average age of 20 years, and were voluntary participants, including 62 females and 18 males, who were in excellent health and had regular studies and lives. There was no history of physical therapy involving muscle electrical stimulation or magnetic stimulation interventions, lumbar cervical injury, magnetic dizziness, antibiotic medication, implantable cardiac pacemaker, stent, metal foreign body and additional devices susceptible to magnetic field over the past 6 months. Before the test, the subjects were informed of the specific arrangements and precautions for the test and signed an informed consent form. The trial was approved by the ethics committee (ethics number: NEU-EC-2023B040S), and registered at the Chinese Clinical Trial Registry (Registration Number: ChiCTR2300078947).

### 2.2 Low-frequency PEMF stimulation instrument

**FIGURE 1:**
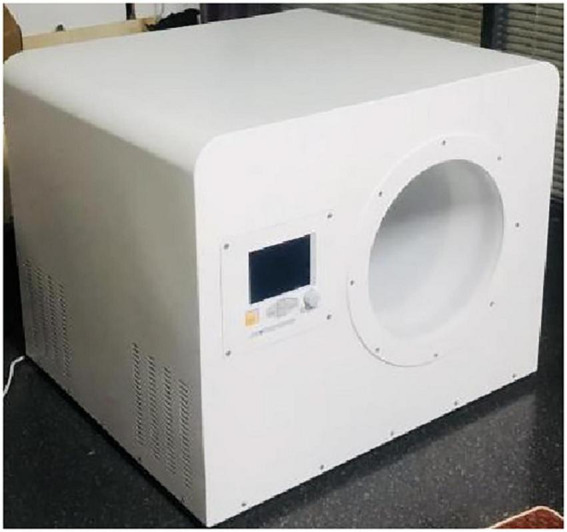
Example of low-frequency pulse magnetic fields instrument.

The instrument comprised two magnetic field intervention devices that we independently developed (patent number: CN115364378A). The instrument structure includes a function generator, power amplifier, and Helmholtz coil equipped with a high-permeability shielding cover. The frequency, duty cycle, high and low potentials, and interval length of the pulse signal were generated and modified using the function generator. The excitation signal was sent to the power amplifier and then amplified and outputted to the Helmholtz coil, where a specified uniform magnetic field was generated. The pulse signal generates a pulsed magnetic field, the interval time is a low potential direct current signal to generate a static magnetic field, and finally, the pulse magnetic field and the static magnetic field are generated alternately. The diameters of the instrument coil were 30 and 60 cm, respectively, with the same depth of 50 cm, which allowed the user to place the entire arm within the range of the magnetic stimulation instrument coil for magnetic stimulation (Figure 1).

### 2.3 Brightness mode ultrasound acquisition

Skeletal muscle morphology parameters were acquired using brightness mode ultrasound imaging (machine model: mindray Eagus R9; 50 mm line array probe L15-3WU; frequency: 7.5 Mhz; depth: 4.5 G; frame rate: 57; dynamic range: 140; speed of sound: 1,580). With the shear wave elastography function, this instrument can measure the elasticity of soft tissues by evaluating the Young’s modulus or calculating the strain ratio of the target tissue relative to its adjacent soft tissue. In addition, the fast, inexpensive, and accessible features make ultrasound elastography an ideal imaging technique for assessing musculoskeletal stiffness, therefore, it is widely used in medicine and sports (27–29).

**FIGURE 2:**
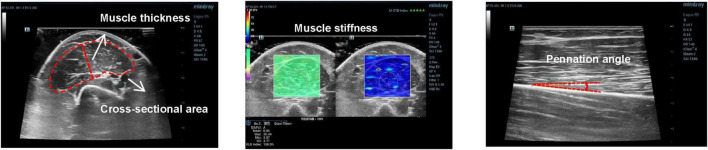
Example of images acquired for pennation angle, muscle thickness, cross-sectional area, and muscle stiffness.

The measurement image was acquired by a physician with 10 years of clinical experience in ultrasound testing, each subject sat in the supine position for 15 min before the acquisition to unify the body fluid and then positioned the collection site at the biceps brachii of the right arm and selected 50% of the distance between the anterior acromion of the acromioclavicular joint, and the elbow fold muscle as the measurement point. First, the ultrasound probe was perpendicular to the muscle fibers, using a transverse position and marking the probe acquisition location with a marker along the edge of the probe (21). Subsequently, images of the muscle thickness and cross-sectional area were acquired at the short head of the biceps brachii, and the muscle stiffness analysis area was delineated using elastic ultrasound. Second, to acquire the pennation angle image, rotate the probe 90° with the transverse position of the probe as the positioning point so that the probe and the muscle fibers were parallel and the longitudinal acquisition position was marked at the edge of the probe. To unify the subsequent measurement and collection positions, the herbal long-term tattoo patch was used to cover the position marked during the first collection process after the first acquisition. Three images were collected for each index, and then without revealing the specific grouping of the collected images, image j software was used to perform image calibration and measurement of muscle thickness, pennation angle, and cross-sectional area by three young physicians with more than 3 years of clinical experience independently. The pennation angle was measured based on the angle formed by the muscle fiber bundle and deep aponeurosis. To minimize the discrepancies arising from the varying locations of muscle bundles in images during the measurement of the pennation angle by different measurers, the following procedure is adopted: First, one physician conducts the initial measurement. Subsequently, the region measured by this physician is calibrated within the image, and the boundaries of the calibrated area are communicated to the other two physicians. This ensures that all three physicians select the pinnate angle at the same location within a single image, thereby reducing measurement variability as much as possible; and the cross-sectional area was measured by the total area delineated along the inside of the fascia as bounded by the fascia outside the short head of the biceps brachii visible in the image. Muscle thickness was obtained by measuring the perpendicular distance from 1/2 of the length of the lower fascia to the upper fascia in the cross-sectional image (Figure 2). Data acquired by three young physicians were averaged to obtain the final results except for the muscle stiffness value, which was obtained using the ultrasonic instrument.

### 2.4 Biceps brachii MVC test

**FIGURE 3:**
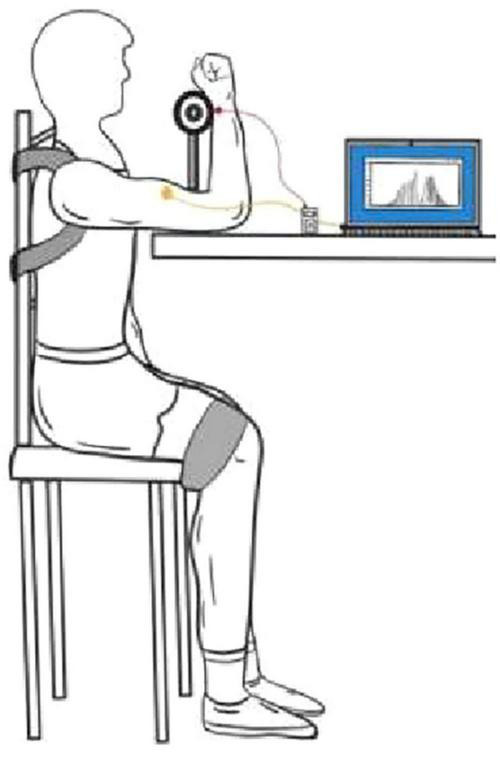
Example of testing the maximum voluntary contraction force of the biceps brachii muscle.

The maximum autonomous biceps force test was performed immediately after the subject’s ultrasound date were collected. The test was performed without informing the tester about the specific subject groupings. The test scheme “The EMG of ABC” was adopted (30). The subject was seated in front of a test bench, approximately 90 cm in height, with the right forearm and upper arm, upper arm and trunk, trunk and thigh, and thigh and calf maintained at 90°, respectively. The shoulders and legs were secured using straps, the left arm was naturally lowered, and the right wrist was held close to a dynamometer. The dynamometer adopts the resistance strain gauge force measurement system with a measuring range of 1–1,000 N (1–100 kg). The dynamometer use computer acquisition software that records the maximum peak and mean force values in real time, with data accurate to 0.1 kg. During the test, as the subjects gradually applied the maximum force, they verbally informed the recorder and then recorded the continuous force with a stopwatch for 5 s, followed by a second test with a 3 min interval. The maximum peak value recorded by the electron dynamometer for both epochs was taken as the MVC maximum for the individual, and the test actions and processes for the left arm were consistent with those of the right arm (Figure 3).

### 2.5 Scheme and process of magnetic field stimulation

**FIGURE 4:**
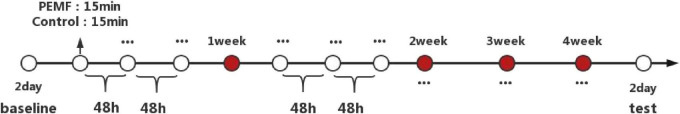
Duration and process of experimental intervention.

Based on the consensus of existing studies, the parameters of our study are as follows: duration: 15 min; interval time: 48 h; frequency: three times a week; total intervention duration: 4 weeks; number of total interventions: 12 times (Figure 4); magnetic intensity: 1.5 mT; magnetic frequency: 3,300 Hz (3, 8, 31). All the parameters followed the international safety standards and the consensus in the field of parameter settings, which is harmless to humans and has no side effects (32–34). The total number of interventions in the control and PEMF groups was the same, except for the instrument being powered on. However, the magnetic field output was turned off in the control group, when the magnetic field stimulation of this device is either activated or deactivated, the subjects will not perceive any differences in heat, vibration, sound, or light, this ensures that the instrument can effectively perform double-blind control. Subjects in both groups were required to eat and rest regularly during the trial, strictly control their physical activity, and avoid any resistance training and sports except for daily activities.

### 2.6 Statistical analysis

Statistical Product and Service Solutions (SPSS) software (version 24.0, IBM, United States) was used to analyze the data, which are expressed as x ± s. The intra-class correlation coefficient is used to calculate the consistency of the image measurements of the three observers. Data on muscle strength and morphological structure related indicators were examined before and after testing for each group. The Shapiro-Wilk test was employed to assess the normality of the data distribution. Paired *t*-tests were used when the data were normally distributed and paired Wilcoxon signed rank-sum tests were used when the data were not normally distributed to verify the differences in the data for each indicator before and after the intervention. To further explore whether the shift in the strength of the left arm in each group was caused by a change in the strength of the right arm after the magnetic field intervention, linear regression analysis was used to investigate the effect relationship. To observe differences between the groups, the rate of change of each index before and after the test was normalized, and the data for the rate of change of all indices in the two groups were tested for normality. If the data did not show significant differences (*p* > 0.05), the differences were directly compared using the independent sample *t*-test if significant (*p* < 0.05). The MannWhitney test statistic was used to study the difference relationship, and all statistical data were collected from *P* < 0.05 were statistically significant.

## 3 Results

### 3.1 Number of subjects, ICC values, pre-test values

**FIGURE 5:**
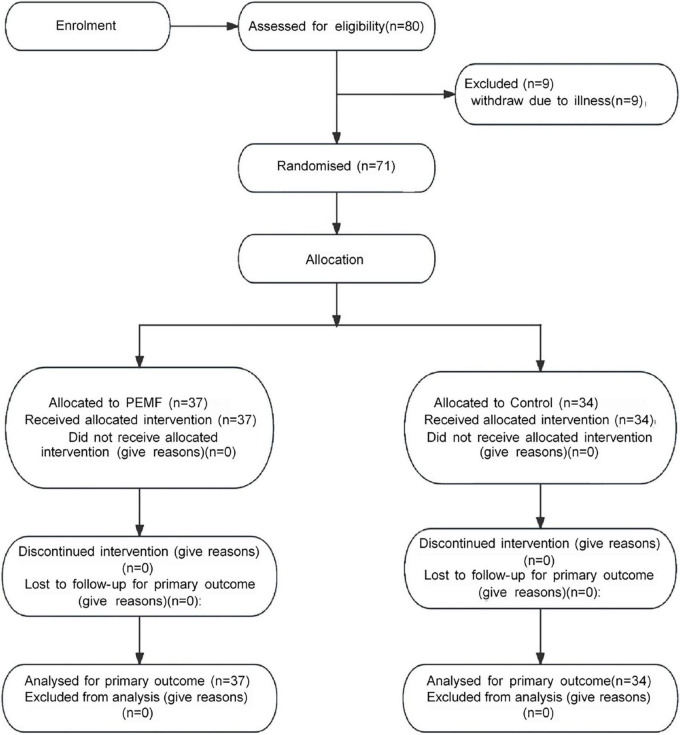
Consolidated standards of reporting trials (CONSORT) flowchart for participant recruitment and allocation.

**FIGURE 6:**
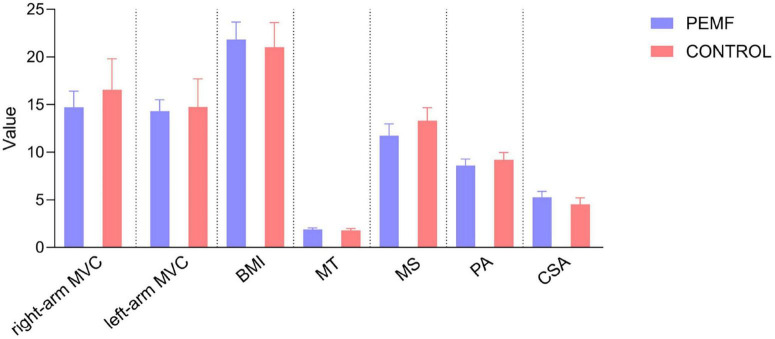
Comparison of pre-test values of muscular strength and muscular structure observation indicators between the two Groups. No significant differences were observed between the two groups in the pre-test values of right-arm maximal voluntary contractivity (MVC) (*p* = 0.227), left-arm MVC (*p* = 0.809), body mass index (BMI) (*p* = 0.991), muscle thickness (*p* = 0.501), muscle stiffness (*p* = 0.243), pennation angle (*p* = 0.234), and cross-sectional area (*p* = 0.055).

Nine subjects withdrew from the study due to illness. Finally, 71 subjects were included in the study data analysis, including 37 subjects (nine males and 28 females) in the PEMF group and 34 subjects (six males and 28 females) in the control group (Figure 5). Three observers measured the thickness, cross-sectional area, and pennation angle of the biceps brachii muscle in 71 participants. Systematic errors were not considered in this study, therefore, so the type of consistency calculation was used. Moreover, the three observers provided the original data rather than the calculated data, so the results of a single metric, ICC (C, 1), were used. The final ICC correlation coefficient values were as follows: muscle thickness (pre-test) 0.976 (95% CI: 0.965–0.984); muscle thickness (post-test) 0.982 (95% CI: 0.973–0.988);cross-sectional area (pre-test) 0.789 (95% CI: 0.706–0.854); cross-sectional area (post-test) 0.783 (95% CI: 0.699–0.850); pennation angle (pre-test) 0.684 (95% CI: 0.575–0.777); pennation angle (post-test) 0.721 (95% CI: 0.621–0.805). These ICC values demonstrate a high level of consistency in the measurement of each morphological index, indicating that the scores provided by the three measurers in this study exhibit excellent reliability (Table 1). No significant differences were observed in the pre-test values of MVC and muscle structure indicators between the two groups, enabling a robust comparison of the experimental intervention effects (Figure 6).

**TABLE 1:** Intraclass correlation coefficient (ICC) values of skeletal muscle structure indicators (*n* = 71).

Morphological parameters	Data collection phase	ICC intra-class correlation coefficient	95% CI
MT	Pre	0.976	0.965∼0.984
	Post	0.982	0.973∼0.988
CSA	Pre	0.789	0.706∼0.854
	Post	0.783	0.699∼0.850
PA	Pre	0.684	0.575∼0.777
	Post	0.721	0.621∼0.805

ICC values greater than 0.75 indicated high consistency, 0.40–0.75 indicated good consistency, and less than 0.4 indicated poor consistency, respectively.

### 3.2 Changes in maximal voluntary contractivity (MVC)

**FIGURE 7:**
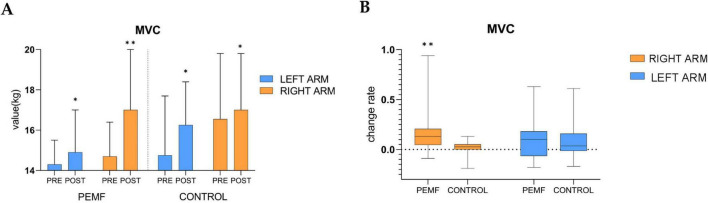
Changes in maximal voluntary contractivity (MVC) within and between groups (*p < 0.05; ^**^p < 0.01). **(A)** The difference between the MVC of the left arm and the right arm pre-test and post-test was analyzed in both groups, low-frequency pulse magnetic fields (PEMF) group (left arm *p* = 0.011, right arm *p* = 0.000),control group (left arm *p* = 0.020, right arm *p* = 0.018). **(B)** After receiving magnetic stimulation intervention, the rate of change in MVC of the left arm (*p* = 0.616) and the right arm (*p* = 0.000) were compared between the two groups.

The MVC values in the left and right arms of both groups showed significant increases compared to the pre-test. After the PEMF stimulation intervention, the strength increase rate of the right arm was 13% (0.0, 0.2), and the increase rate of the left arm was 10% (−0.1, 0.2). In the control group, the right arm increase rate was 2.5% (0.0, 0.1), and the left arm increase rate was 3.5% (−0.0, 0.2). The shift in intensity in this group may be related to the fact that the test movement is more proficient in generating force, as confirmed by follow-up communications with subjects after the test, therefore, the shift in intensity in the control group was a normal fluctuation and comparisons between the two groups showed that the elevation of the right arm in the PEMF group was significantly higher than that in the control group (*p* = 0.000), while there was no significant difference in the elevation of the left arm between the two groups (*p* = 0.616) (Figure 7).

### 3.3 Changes in body mass index (BMI)

**FIGURE 8:**
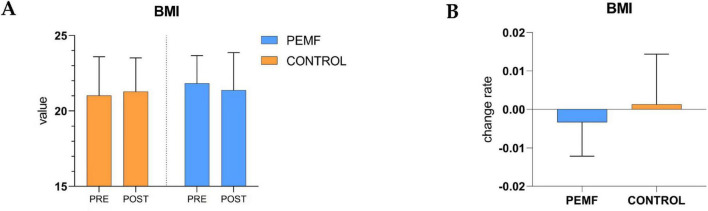
Changes in in body mass index within and between groups. **(A)** The difference of body mass index body mass index (BMI) values between pre-test and post-test in the two groups of subjects was analyzed, low-frequency pulse magnetic fields (PEMF) group (*p* = 0.484), control group (*p* = 0.281). **(B)** The rate of change in BMI values before and after testing was compared between the two groups (*p* = 0.221).

There was no significant difference in BMI between the two groups during the pre-test and post-test phases, and there was no significant difference in the change rate between the two groups, PEMF group (*p* = 0.484), control group (*p* = 0.281). However, there was a downward trend in BMI in the PEMF group and an upward trend in the control group (Figure 8).

### 3.4 Morphological changes in skeletal muscles

**FIGURE 9:**
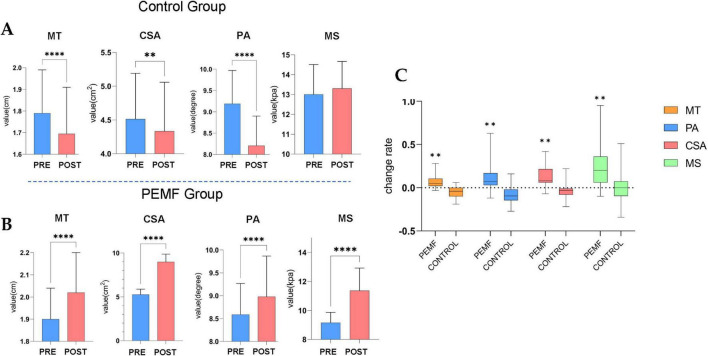
Morphological changes in skeletal muscles (^****^p = 0.000; ^**^p < 0.01). **(A)** In the control group, the muscle thickness (MT) (*p* = 0.000), pennation angle (PA) (*p* = 0.000) and cross-sectional area (CSA) (*p* = 0.008) values were significantly decreased. **(B)** The difference of skeletal muscle structure index between pre-test and post-test in low-frequency pulse magnetic fields (PEMF) group, MT (*p* = 0.000), PA (*p* = 0.000), CSA (*p* = 0.000), and muscle stiffness (MS) (*p* = 0.000) values were significantly increased. **(C)** The rate of change in skeletal muscle structural indicators was compared between the two groups of pre-test and post-test. The significance *P*-values of the four structural indicators among groups were all ^**^p < 0.01.

After 4 weeks of PEMF stimulation intervention, all four parameters of the biceps brachii muscle in the PEMF group showed significant increases compared to the pre-test. Muscle thickness increased by 5% (0.0, 0.1), pinna angle increased by 7% (0.0, 0.2), cross-sectional area increased by 8% (0.1, 0.2), and muscle stiffness increased by 20% (0.1, 0.4). After 4 weeks, the control group strictly controlled the movement of skeletal muscle morphology and presented significant attenuation condition, this group of decreases in muscle thickness was 4% (−0.1, 0.0), the pennation angle decline rate was 9.5% (−0.1, −0.0), the cross-sectional area decline rate was 3% (−0.1, −0.0), and muscle hardness compared to the pre-test although it did not change significantly, but the overall average decreased. By comparing the effects of the two intervention methods on the morphology of skeletal muscle, it was found that the four indices of muscle morphology in the PEMF group were significantly higher than those in the control group, which proved that the low-frequency pulsed magnetic fields had a significant promoting effect on the morphology of the skeletal muscle (Figure 9).

### 3.5 Impact of intervention arm on strength of non-intervention arm

**FIGURE 10:**
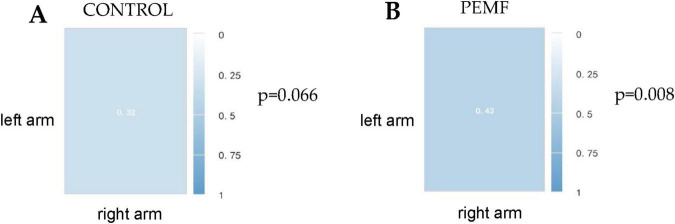
Spearman correlation coefficient between left and right arm strength change rate in the two groups. **(A)** In the control group, the correlation between the rate of strength improvement in the left arm and that in the right arm was 0.32 (*p* = 0.066). **(B)** In the low-frequency pulse magnetic fields (PEMF) group, the correlation between left arm strength change rate and right arm strength change rate was 0.43 (*p* = 0.008).

The correlation between the right arm strength change rate and the left arm strength change rate in the PEMF group was 0.429 and was significance at the 0.01 level (*p* = 0.008), thus indicating a significant positive correlation between the right arm strength change rate and the left arm strength change rate. In the control group, the correlation between the right arm strength change rate and the left arm strength change rate was 0.319, and the *P*-value was 0.066 > 0.05, showing that there was no correlation between the change rate in the right arm and that in the left arm (Figure 10).

**FIGURE 11:**
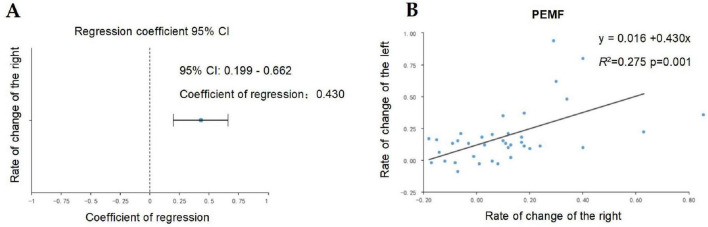
Linear regression analysis of strength improvement rates between right and left arms in the low-frequency pulse magnetic fields (PEMF) group. **(A)** The regression coefficient value of the right arm strength change rate is 0.430 (95% CI: 0.199–0.662, t = 3.641, *p* = 0.001 < 0.01), indicating that the right arm strength change rate has a significant positive influence on the left arm change rate. **(B)** The model formula is: left arm change rate = 0.016 + 0.430 × right arm change rate. The R-squared value of the model is 0.275, meaning that the right arm change rate under PEMF intervention can explain 27.5% of the variation in the left arm change rate, suggesting that the right arm change rate will definitely have an impact on the left arm improvement rate.

Because there was a significant positive correlation between the strength increase rate of the two arms in the PEMF group, and the data of the strength increase rate of the left arm were normally distributed (statistic W = 0.956, *p* = 0.148), the strength increase rate of the right arm in the PEMF group was used as the independent variable, and the strength increase rate of the left arm was used as the dependent variable for linear regression analysis, The model formula was as follows: the left arm change rate = 0.016 + 0.430 × right arm change rate, and the R square value of the model was 0.275, which meant that the strength change rate could explain 27.5% of the variation in the left arm change rate. F-test showed that the model passed the F test (F = 13.259, *p* = 0.001 < 0.05), that is, the right arm strength improvement rate in the PEMF group had an effect on the left arm strength improvement rate (Figure 11).

## 4 Discussion

Our results showed that after 4 weeks of chronic exposure to low-frequency PEMF, the MVC of the PEMF and control groups increased significantly, at a rate of 13% and 2.5%, respectively. The increased amplitude in the PEMF group was more consistent with the results of our previous study (9). The fluctuation change of the force in the control group could be attributed to the interference caused by the increased proficiency of the subjects in the test, which was further confirmed by the subjects themselves in the control groups indicating that they were more adaptable to force action than before the test. The gain of skeletal muscle structure morphology is bound to promote improving strength (13). Our study demonstrated a significant increase in the four morphological indices of muscle cross-sectional area, pennation angle, muscle thickness, and muscle stiffness, which played a crucial role in the strength improvement in the PEMF group. In contrast, the muscle morphology indicators of the control group decreased significantly under the control of exercise avoidance for 4 weeks. The decline in the structural indicators in the control group may be related to the strict exercise restrictions in the experiment and more female subjects were involved. Previous studies have suggested that skeletal muscle strength and morphological structure decay faster in women than in men after muscle unloading (35). In a resistance training study, eccentric training had a better effect on muscle morphological structure, and we observed that although PEMF stimulation significantly improved skeletal muscle morphology, it was slightly lower than that of 4 weeks of eccentric resistance training on muscle tissue (36). The significant improvement in morphological indices in this test supported the change in the MVC of the tested skeletal muscle in the PEMF group. It is generally believed that the cross-sectional area of muscles is related to the number of parallel muscle segments and sarcoplasmic volume and affects the force production ability of muscles (37, 38). A larger muscle area produces a greater force, which has always been the focus of skeletal muscle function research and is the primary basis for force changes (12, 39). However, the ability of torque to produce force at the angle of limb joints should also be considered when evaluating the effect of human strength. The pennation angle is the angle between the direction of muscle fibers and the force of muscles and is often used as the primary indicator of benign changes in muscle structure in sports training (40). The pennation angle is highly correlated with the number of bundle’s of muscle fibers and thickness (41, 42). In our test, we also observed that muscle thickness and cross-sectional area indicators increased with an increase in the pennation angle. Another significant change is muscle stiffness, which has been shown to increase with the increases in muscle mass, thickness, and strength (43). In healthy individuals, increased muscle thickness promotes benign changes in the internal muscle structure and composition, which results in increased resting muscle tone and enhanced muscle stiffness (44). Another study assessing the muscle stiffness of healthy individuals using shear wave ultrasonic elastic technology showed that, the stiffness of the biceps brachii increased with a reduction in BMI. Franco-obregón proposed that magnetic stimulation can exert a benign effect on body fat metabolism, reduce body fat content, and support the improvement of BMI indicators. The current study found that the BMI of the PEMF group indicate a downward trend and muscle stiffness increased. In contrast, the BMI of control group demonstrate an upward trend and decreased muscle stiffness, which is consistent with existing findings.

Indeed, morphological changes have been observed in muscle hypertrophy following PEMF stimulation. However, we cannot determine whether the hypertrophy change is attributable to sarcoplasmic hypertrophy or myofibrillar hypertrophy, as both could lead to improved skeletal muscle morphological indicators (14, 38, 45). Although sarcoplasmic hypertrophy can gain muscle strength (46), myofibrillar hypertrophy could fundamentally change the myofiber contractile unit, which further supports the improvement of strength and the subsequent increase in the reserve capacity of intensive exercise (47). Therefore, from the perspective of muscle health and exercise capacity promotion, we prefer PEMF stimulation technology to produce myofibril hypertrophy. Changes in muscle composition cannot be analyzed owing to the limitations of ultrasound image technology (25). Therefore, we could not determine the hypertrophy pattern, and histological staining could detect the specific pattern of muscle hypertrophy caused by PEMF stimulation.

According to Alfredo Franco-obregón’s explanation, the mechanism by which magnetic fields may cause hypertrophy in muscle tissue is that a specific low-frequency PEMF can stimulate TRPC 1 calcium ion channels and activate downstream calcineurin transduction (CaN), which is considered to be an essential signaling molecule for skeletal muscle remodeling that is widely involved in skeletal muscle differentiation, fiber transformation, and other processes, and plays a vital role in Ca^2+^ mediated cellular responses (48). The transcription of slow twitch phenotypic protein was increased through the synergistic effect of the nuclear factor of activated T cells (NFAT) and myocyte enhancement factor. Dephosphorylated by CaN, NFAT can translocate to the nucleus and bind to corresponding molecules to promote specific gene expression, activate histone acetyltransferase PCAF, enhance the expression of MyoD, and promote protein synthesis and myogenesis, so that muscle tissue can grow and adapt to changes (3). Furthermore, based on the specificity of low-frequency PEMF-mediated TRPC 1 channel activation, we believe that magnetic stimulation may also promote tissue morphological improvement through satellite cells and activation of the PI3K/Akt/mTOR/p70S6K pathway, which are considered to be the primary forms of muscle fiber growth and protein synthesis caused by resistance training and studies have confirmed the critical role and specific mechanism of the TRPC 1 channel regarding these two aspects, as well as the promotion effect on muscle regeneration (49, 50). TRPC1 can stimulate myogenesis independently without the PI3K/Akt pathway. TRPC1 interacts with I-mfa, an inhibitor of the myogenic family, which competes with the binding of I-mfa to myogenic proteins and releases active myogenic proteins that trigger myogenesis (51). Therefore, it is a very worthy and exciting topic that if TRPC 1 channels can be activated by magnetic field stimulation and the co-activation of the above mechanisms can be achieved, this will bring a revolutionary application strategy for the field of sports training. The disturbing effects of endurance and resistance training on muscle hypertrophy and muscle strength performance have long plagued sports practitioners.

Resistance training induces skeletal muscle adaptation mainly by activating the mTOR signaling pathway (52, 53). In contrast, endurance training activates the calmodulin-dependent protein kinase CaMK and upregulates PGC-1α expression by releasing Ca^2+^ in the sarcoplasmic reticulum, enhancing mitochondrial biosynthesis (54). The principal contradiction between these two training methods is the generation of specific molecules and the activation and inactivation of different cell signaling pathways (55). According to Alfredo Franco-obregón’s study, the molecular mechanism by which magnetic-induced muscle improvement is enhanced in vitro myogenesis and mitochodriogenesis by activating the PGC-1α transcriptionally regulated calcium-mitochondrial axis through the TRPC 1 channel is consistent with the mechanism of endurance training on skeletal muscle. According to the theory of simultaneous interference effects, the maximum strength and muscle hypertrophy induced by magnetic stimulation should be weak. However, we observed simultaneous improvements in maximum strength, strength endurance, explosiveness, and muscle morphology in our current and previous studies without simultaneous interference effects caused by magnetic stimulation (9–11). Some scholars have also proposed that simultaneous training does not induce an interference effect on skeletal muscle hypertrophy, and the key influencing factors in regulating the two training methods leading to muscle hypertrophy are the training load and the duration of simultaneous training (56–58). The stimulation intensity and frequency of different training sessions determine the presence of an interference effect. As for the data of our current study, whether the result is caused by the higher magnetic field parameters, higher stimulation frequency, or the frequency and strength of the magnetic field in this test are more in line with the optimal parameter combination of muscle hypertrophy and endurance improvement. This problem will be investigated in depth in future studies, and we hope that our study will attract the attention of researchers in this field to use this new magnetic field parameter to solve the interference effect problem.

In addition to the supporting elements of muscle structure, energy supply and nerve conduction level of muscle tissue are also important factors that could contribute to the improvement in strength observed in our trial, and low-frequency PEMF may support the above two factors. The energy support core of skeletal muscle strength is the supply capacity of ATP. Analysis based on the existing mechanism showed that low-frequency PEMF could induce TRPC 1 calcium ion channel, cause an increase in calcium ions in the cytoplasm, enhance the activity of calcineurin, catalyze NFAT dephosphorylation, promote NFAT translocation to the nucleus of muscle cells, and activate peroxisome proliferator-activated by NFATC 1 and myocyte specific enhancer factor 2 to activate PGC-1α (8). PGC-1α, one of the key signaling molecules for energy metabolism in skeletal muscle, is a transcriptional activator of the nuclear receptor family, is widely involved in multiple metabolic pathways such as mitochondrial biosynthesis, and is a crucial regulator of mitochondrial biosynthesis. PGC-1α acts as an effective controller of mitochondrial function and energy homeostasis and functions synergistically with calcineurin/NFAT catalytic cascade to enhance mitochondrial respiration (59, 60). The improved mitochondrial respiratory function ensures rapid ATP supply in muscle cells and maintenance of intracellular ATP content. This is particularly important during maximum voluntary contraction at the ultimate strength of short-term rapid mobilization of muscle fibers (61).

In the state of extreme strength isometric contraction, the conduction ability of nerve signals sent by the central nervous system plays the role of rapid mobilization of motor units, especially the transmembrane conduction ability of action units, and Ca^2+^ plays a vital role in this process (12, 62). Low-frequency PEMF stimulation as an exogenous stimulation can activate TRPC 1 calcium channel, increasing the content of Ca^2+^ in muscle cell membranes and increasing the excitability of myocyte membrane, thereby making the nerve conduction process more efficient and ensuring the rapid and continuous conduction of nerve signals (63). A previous study found that a high degree of muscle activation can still be maintained in skeletal muscle fatigue, and the spectrum of electromyograms shifts significantly to the right. The benign improvement of the surface electromyogram signal can indirectly confirm the support effect of high steady-state Ca^2+^ on action potential transmission in muscle cells and promote maximum strength increase (9).

It is worth mentioning that when observing the changes in left and right arm strength of subjects in different cohorts, it was found that with an increase in arm strength on the intervention side of the PEMF group, the strength of the contralateral arm also increased with a significant positive relationship with the intervention arm. However, these results were not observed for the control group. Franco-obregón observed the contralateral effect of this technique on the non-intervening arm in a previous study (6, 7). Our current clinical trial also observed this phenomenon which has a wide range of applications in medical rehabilitation or sports training, when the injured limb is inconvenient to move during the recovery period for disabled people or athletes because it can be used to improve muscle strength on the non-injured side and reduce the impact caused by mobility limitation.

## 5 Conclusion

The current clinical trial showed that in healthy individuals aged 20 years, after 4 weeks of chronic low-frequency PEMF (1.5 mT, 3,300 Hz) exposure, the maximum voluntary contractility of the biceps brachii was significantly improved, and the contralateral effects were verified. Meanwhile, significantly improved muscle thickness, cross-sectional area, pennation angle, and stiffness of the intervention arm were observed, which provides solid evidence for applying this technology as a choice or medical strategy for muscle improvement.

## 6 Innovations and limitations

To our knowledge, this is the first study of low-frequency PEMF-induced muscle morphological changes based on improving muscle tissue function by activating the PGC-1α transcriptionally regulated calcium-mitochondrial axis through the TRPC 1 channel, which provides valuable information for applying this technology as a sports and medical strategy to improve muscle function.

Furthermore, together with previous studies, the current clinical trial found simultaneous improvements in maximum strength, strength endurance, explosiveness, and muscle morphology, and no simultaneous interference effects were caused by PEMF stimulation in the exercise training process in human trials. We hope that this discovery will draw the attention of researchers in this field and provide important directions for future research.

Although we observed muscle hypertrophy caused by PEMF stimulation, it is not clear whether sarcoplasmic hypertrophy or myofibril hypertrophy contributes to this phenotype because of the limitations of the ultrasound imaging technology. Therefore, the specific pattern of muscle hypertrophy can be analyzed using histological staining technology in future studies. Another limitation was that the ultrasound image of skeletal muscle morphology was not acquired from the non-interventional arm in our trial. Therefore, the discovery of the contralateral effect was based only on observed changes in arm strength, which lacked morphological changes. As for the subjects, women whose muscle mass and strength were weaker than those of men comprised a large portion of the subjects in this trial, and the growth and decay rate of muscle hypertrophy in women was faster than that of men after physical stimulation (64). It has been shown that low-frequency PEMF stimulation has a better effect on individuals with weak muscle mass, which may be one of the reasons for the significant increase in strength and muscle morphology in the PEMF group and attenuation of strength and muscle morphology in the control group. Further studies are required to investigate the effects of this technique on the changes in male muscle morphology.

In addition, considering the age composition, the number of subjects enrolled in this trial was 20. Most of them are women and lack adolescents, middle-aged and elderly subjects because the muscle content of adolescents, middle-aged and older adults is lower than that in adults, and the expression of TRPC1 in muscle tissue is also lower than that of adults. Therefore further clinical trials are needed to verify whether the current PEMF stimulation scheme could obtain the same results among adolescents and middle-aged and elderly individuals.

## Data Availability

The original contributions presented in this study are included in this article/supplementary material, further inquiries can be directed to the corresponding author.
